# Classification of Tea Aromas Using Multi-Nanoparticle Based Chemiresistor Arrays

**DOI:** 10.3390/s19112547

**Published:** 2019-06-04

**Authors:** Tuo Gao, Yongchen Wang, Chengwu Zhang, Zachariah A. Pittman, Alexandra M. Oliveira, Kan Fu, Jing Zhao, Ranjan Srivastava, Brian G. Willis

**Affiliations:** 1 Department of Chemical and Biomolecular Engineering, University of Connecticut, Storrs, CT 06269, USA; tuo.gao@uconn.edu (T.G.); chengwu.zhang@uconn.edu (C.Z.); zachariah.pittman@uconn.edu (Z.A.P.); alexandra.oliveira@uconn.edu (A.M.O.); ranjan.srivastava@uconn.edu (R.S.); 2 Department of Chemistry, University of Connecticut, Storrs, CT 06269, USA; yongchen.wang@uconn.edu (Y.W.); jing.zhao@uconn.edu (J.Z.); 3 Department of Materials Science and Engineering, University of Connecticut, Storrs, CT 06269, USA; kan.fu@uconn.edu; 4 Institute of Materials Science, University of Connecticut, Storrs, CT 06269, USA

**Keywords:** tea aroma sensing, gold nanoparticles (AuNPs), chemiresistor array, linear discriminant analysis (LDA), pattern recognition

## Abstract

Nanoparticle based chemical sensor arrays with four types of organo-functionalized gold nanoparticles (AuNPs) were introduced to classify 35 different teas, including black teas, green teas, and herbal teas. Integrated sensor arrays were made using microfabrication methods including photolithography and lift-off processing. Different types of nanoparticle solutions were drop-cast on separate active regions of each sensor chip. Sensor responses, expressed as the ratio of resistance change to baseline resistance (Δ*R*/*R_0_*), were used as input data to discriminate different aromas by statistical analysis using multivariate techniques and machine learning algorithms. With five-fold cross validation, linear discriminant analysis (LDA) gave 99% accuracy for classification of all 35 teas, and 98% and 100% accuracy for separate datasets of herbal teas, and black and green teas, respectively. We find that classification accuracy improves significantly by using multiple types of nanoparticles compared to single type nanoparticle arrays. The results suggest a promising approach to monitor the freshness and quality of tea products.

## 1. Introduction

As one of the most popular beverages, tea is consumed by hundreds of millions of people worldwide [1]. Known for its health benefits such as energizing, relieving stress, strengthening the immune system, and alleviating digestive problems [2,3,4], tea has been intensively studied for its effects related to human health [5,6,7]. Green tea and black tea are harvested directly from the leaves of tea plants, *Camellia sinensis*, which contain many polyphenols that are natural antioxidants [1]. Herbal teas, on the other hand, are mixtures of caffeine-free leaves, seeds, spices, and plant roots that give a unique aroma and taste. Some of the most common ingredients include chamomile, cinnamon, ginseng, ginger, hibiscus, peppermint, and rose hip [2]. Similar to green tea and black tea, herbal tea also provides many medicinal properties, such as destressing and certain disease prevention [2,8,9]. One key property that distinguishes tea products is aroma, which is also a key factor for consumer taste. Depending on the interactions between constituted ingredients and aroma compounds, each tea product has a complex odor and smells differently [10]. Furthermore, aroma is also related to freshness and quality of food products. For example, a change in odor can be related to aging or staling of a product. Historically, human experts are typically trained to evaluate the quality of tea products. They taste and smell teas to assign quality grades. However, this can be subjective and expensive. It is interesting to consider whether chemical sensors can provide cost-effective alternatives to recognize and classify these complex odors with high accuracy.

Researchers have investigated different chemical and biological sensing systems based on fluorescence [11], surface enhanced Raman scattering (SERS) [12], wavelet energy [13], and quartz crystal microbalances (QCM) [14]. These various sensing strategies have shown promising applications with good selectivity and specificity [11,12,13,14], especially under physiological conditions [15]. On the other hand, to detect and differentiate many different analytes, a less specific and weakly selective sensor is desired to record overall sensing patterns. In the past 10 years, chemiresistors have attracted attention due to the capability to respond to a wide range of analytes. For example, chemiresistor sensor arrays fabricated with metal oxides have been reported for sensing tea aromas [16,17]. Chemiresistors are electrical devices that sorb vapor analytes into sensing materials, causing resistance changes that transduce chemical events into electrical signals, which can be processed with machine learning algorithms to recognize sensing patterns [18]. Therefore, to detect and classify many tea aromas, sensor materials with weak selectivity and broad response profiles are useful.

Compared with traditional chemiresistive sensing elements such as metal oxides or catalytic metals [18], monolayer protected nanoparticles show more versatile capabilities due to their straightforward synthesis methods and tunable properties, including the sizes and shapes of the metal cores, and the chemical structure of the self-assembled monolayer shells [16,18]. Consequently, nanoparticle-based sensors enable array-based selectivity for pattern recognition of many types of analytes [18,19] and have been applied in areas such as environmental testing [20], health monitoring [21], and public safety [12]. The developments of nanoparticle-based chemical sensors date back to 1998, when Wohltjen and Snow first introduced a core-shell sensing material using nanoparticles with self-assembled monolayers for organic vapor detection [22]. Their study demonstrated fast and reversible responses for toluene and tetrachloroethylene using 2-nm octanethiol-capped gold nanoparticles (AuNPs) [22]. Since then, intensive studies have been carried out focusing on sensor performance improvement. Functional layers such as amines [23,24,25], biomolecules [26,27], and polymers [28,29] have been reported as capping agents in metal nanoparticle chemiresistors to classify room temperature gases or organic vapors with good accuracy. Still, one challenge of metal nanoparticle-based chemiresistors is to optimize the number of chemiresistors and monolayer types, so that devices not only provide sufficient resolving power to differentiate complex odors, but also give reliable performance over time. One possible solution is to assemble many different types of sensing chemistries into a single sensor chip [30]. Each array provides a fingerprint (odor print) that can be used to statistically characterize and separate analytes.

In this study, we present a nanoparticle-based electronic nose that distinguishes 35 different teas from three different categories (black tea, green tea, and herbal tea). Sensor electrodes were fabricated using microfabrication methods, and then assembled with four types of nanoparticles as the sensing elements, including a pyridine derivative (DMAP, 4-dimethylaminopyridine), a long-chain alkyl amine (ODA, octadecylamine), a bifunctional alkyl thiol (3-MPA, 3-mercaptopropionic acid), and a bifunctional aromatic thiol (4-ATP, 4-aminothiophenol). These monolayer protected AuNPs were chosen based on earlier success in organic vapor sensing [30].

During AuNP synthesis, organic molecules adsorb onto nanoparticle surfaces and form self-assembled monolayers (SAMs) [31]. The nanoparticle solutions were drop-cast at each corner of a sensor chip to assemble working chemiresistors. To evaluate the performance of the electronic nose system, different statistical analysis methods were utilized, including linear discriminant analysis (LDA), support vector machine (SVM), k-nearest neighbors (KNN), and random forest (RF). For each tea aroma, individual sensor responses were input as variables in a data matrix. With five-fold cross-validation, 100% and 97.7% classification accuracy was achieved for black and green teas, and herbal teas, respectively. The results also show that by incorporating multiple sensing chemistries, the classification accuracy improves, compared with devices assembled with only one sensing material. An additional five-day experiment was conducted to assess the short-term stability of the sensors, which maintained above 90% accuracy. The results demonstrate that nanoparticle-based chemiresistor arrays can be suitable candidates for tea aroma sensing and classification, which may be useful for evaluation of the quality and freshness of tea products.

## 2. Materials and Methods

### 2.1. Chemicals and Tea Analytes

Gold chloride trihydrate (HAuCl_4_·3H_2_O), tetraoctylammonium bromide (TOAB), sodium borohydride, sodium carbonate, 4-(dimethylamino)pyridine (DMAP), octadecylamine (ODA), 3-mercaptopropionic acid (MPA), 4-aminothiophenol (ATP), and (3-mercaptopropyl)triethoxysilane (3-MPTES) were purchased from Sigma-Aldrich (St. Louis, MO, USA). Ethanol, toluene, and sulfuric acid were purchased from Fisher Scientific (Hampton, NH, USA). All chemicals were used as received from the manufacturer. Milli-Q deionized water was used for aqueous nanoparticle solution preparation (MilliporeSigma, Burlington, MA, USA). Oxidized silicon wafers with 300 nm SiO_2_ were purchased from University Wafer (South Boston, MA, USA).

Sensing measurements were performed with 35 tea samples with different aromas, including eight black teas, five green teas, and 22 herbal teas, purchased from Twinings (Andover, UK), Celestial Seasonings (Boulder, CO, USA), and Solstice Tea Traders (Corbin, KY, USA). The detailed information of the 35 tea samples are shown in Table 1. All tea samples were collected and used as received.

### 2.2. Synthesis of Nanoparticles

Four types of monolayer protected gold nanoparticles (AuNPs) were used for sensing experiments. DMAP-AuNPs were synthesized following a method reported by Hubble and coworkers with an average size of 5 nm [20]. Gold nanoparticles were first synthesized using the Brust method [32] and followed by phase transfer into an aqueous phase [33]. ODA-AuNP, MPA-AuNP, and ATP-AuNP with approximate size range 10–20 nm were prepared using an approach developed by Chen et al. [34] with minor modifications. ODA (4 mmol) and HAuCl_4_·3H_2_O (0.05 mmol) were added into a 25-mL three-neck flask with a magnetic stir bar. The system was degassed by nitrogen gas to remove dissolved oxygen. The temperature was increased to 140 °C and kept for 20 min. Once the reaction was complete, a mixture of ethanol and toluene was added to remove excess reactants and surfactants. The nanoparticles were dissolved in ethanol for further use. For MPA-AuNP and ATP-AuNP synthesis, an equimolar of MPA and ATP were added to the reaction mixture, respectively, to complete ligand exchange reactions from ODA. Thiol-based chemistry is commonly used for monolayer formation on AuNPs and provides good sensing performance with long-term stability [35,36]. Other possible linker chemistries include diazonium compounds, which also provide robust core-shell structures [37,38,39]. Transmission electron microscopic imaging (Talos F200X TEM, Thermo Fisher Scientific, Waltham, MA, USA) was performed to measure the morphology and sizes of as-synthesized gold nanoparticles.

### 2.3. Fabrication of Sensor Arrays

Chemiresistor devices with 5 μm wide electrodes and 2 μm electrode separations were fabricated using standard photolithography and liftoff processes on 4 inch silicon wafer substrates, following a previously reported method [30]. Each sensor chip had 48 individually addressable sensor elements that were separated into four groups at each corner, consisting of a 4960 μm^2^ total active sensing area. Photolithography was performed using a maskless aligner (Heidelberg Instruments, Heidelberg, Germany) that defined the devices and their electrical contacts. Gold microelectrodes and electrical contacts were obtained by electron-beam evaporation of 200 nm Au with a 10 nm Ti adhesion layer. The electrodes were obtained after liftoff processing. Subsequently, wafers were diced into individual chips 15.9 × 15.9 mm^2^ in size. Before nanoparticle deposition, sensor chips were immersed in a 3-MPTES solution (2% v/v in toluene) overnight at room temperature for thiol-decoration on SiO_2_ surface areas. The chip was then washed with toluene and dried by nitrogen, followed by a 2-hour bake at 110 °C to improve the coupling capability of 3-MPTES [40]. 

### 2.4. Aroma Sensing Experiments

Gold nanoparticle solutions were deposited onto the microelectrode regions of a sensor chip with direct drop-casting. A 2 μL droplet of each type of AuNP was placed at a corner of a sensor chip using a micropipette. The droplets covered the entire active sensing region of the devices. Once the solvent evaporated, the surface of the chip was cleaned by a stream of dry nitrogen gas (99.999% purity). Scanning electron microscope analysis (Verios 460L SEM, Thermo Fisher Scientific, Waltham, MA, USA) was conducted to verify nanoparticle assembly at the microelectrodes.

**Figure 1:**
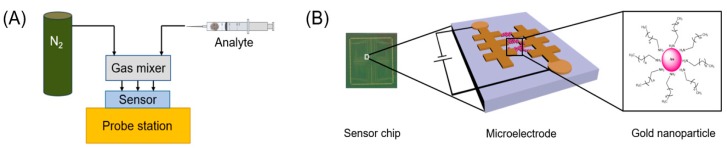
(**A**) Sensing experimental setup with the electronic nose sensor chip; (**B**) schematic illustration of the sensor chip, microelectrode features, and gold nanoparticle core-shell structure.

Room temperature sensing experiments were performed using a probe station (Rucker & Kolls, Mountain View, CA, USA) with a custom-designed probe card (Wentworth Laboratories, Brookfield, CT, USA) for electrical contacts. Figure 1 shows a schematic illustration of the electronic nose system. High speed data acquisition was made using a switch matrix/multimeter system (Keithley, Cleveland, OH, USA) that was connected to the probe card. Dry tea leaves and powders (average 0.40 ± 0.01 g) were extracted from their original packages and transferred into 20-mL syringes (BD, Franklin Lakes, NJ, USA). The tea samples were stored in syringes overnight to allow tea aromas to equilibrate. Tea aroma samples in air were delivered to a custom-made Teflon gas mixer by a syringe pump (Cole-Parmer, Vernon Hills, IL, USA). A dry nitrogen stream was used as a mixing/purge gas to combine with the aromas inside the gas mixer and to clear the sensor after each syringe pulse.

Real-time resistance data were obtained from the switch matrix/multimeter system, using a constant-current sourcing method for *R_0_* ≤ 1 MΩ or a ratiometric method for *R_0_* > 1 MΩ. To extract sensing data, a custom MATLAB program was used to process raw data and calculate sensor responses as Δ*R*/*R_0_*, where *R_0_* is the baseline resistance and Δ*R* is the deviation from baseline resistance due to tea aroma interactions with sensor elements. 

### 2.5. Data Analysis

For each tea aroma sample, a total of eight replicate measurements (pulses of vapor) were performed on all 48 devices simultaneously. The sensor responses were extracted into a 280 × 48 data matrix that was used for statistical analysis. The rows represent individual measurements, and the columns represent the sensor element responses (Δ*R*/*R_0_*). Principal component analysis (PCA) was conducted to visualize grouping within each aroma and separation between aromas. To evaluate the discrimination performance of the electronic nose array, cross-validation was conducted along with linear discrimination analysis (LDA), support vector machine (SVM), k-nearest neighbors (KNN), and random forest (RF) in order to calculate the classification accuracy. Data analysis and pattern recognition were done using the MATLAB Classification Learner Toolbox for LDA, SVM, and KNN, and coding with the programming language Julia for RF.

## 3. Results

### 3.1. Sensor Response Profiles

**Figure 2:**
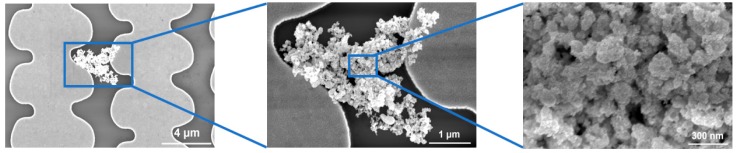
SEM images after drop-casting deposition of 3-mercaptopropionic acid functionalized gold nanoparticles (MPA-AuNP).

Figure 2 shows scanning electron microscope images of micro-junctions assembled with MPA-functionalized gold nanoparticles. The average baseline resistance for MPA-AuNP (15-nm average size) chemiresistors was 41.0 ± 21.8 MΩ. By using direct drop-casting, clusters of AuNP sensing elements covered adjacent electrodes to form a closed electrical circuit. The cluster formation ensured that electron transport occurs between nanoparticles. The low coverage of the electrodes in the figure was typical of the experiments, and the curvature of the microelectrodes is due to the resolution limit of the maskless writer.

Before conducting sensing experiments, the baseline resistance of each of the 48 individual devices was examined. For devices with baseline resistance *R_0_* < 10^3^ Ω, it is likely that large agglomerations of nanoparticles formed and covered the electrode junctions, or that layers contain partially sintered particles [20,30,41]. These devices usually have less than 0.01% change of resistance for interactions with vapor analytes and are not useful sensor elements. On the other hand, devices with high baseline resistance (*R_0_* > 10^8^ Ω) are also removed from the analysis. These high resistance chemiresistors may have incomplete nanoparticle films, and sensing responses are typically noisy (low S/N ratio). Therefore, only devices with baseline resistance in the range of 10^3^ Ω ≤ *R_0_* ≤ 10^8^ Ω were included in sensor performance evaluation.

**Figure 3:**
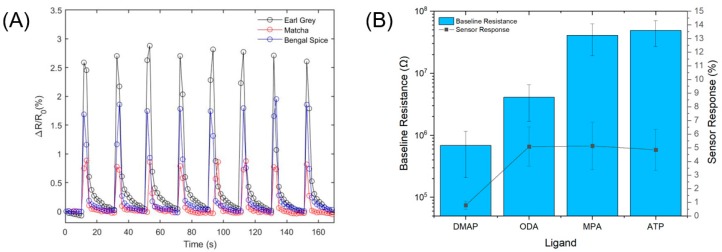
(**A**) Real-time sensor response profiles for Earl Grey (black tea), Matcha (green tea), and Bengal Spice^®^ (herbal tea) for one sensor element with DMAP-AuNP; (**B**) Baseline resistance and sensor response variation among four types of organo-capped gold nanoparticles toward Black Cherry Berry (herbal tea).

The sensor chip was first measured under 0.5 SCFH (236 SCCM) of purge gas flow to reach a stable baseline resistance and then exposed to eight sequential 2.5 s pulses (3.3 mL per pulse) of a tea aroma. The total experimental duration was 170 s for each tea analyte. Each peak represents a sampling period that includes a pulse phase and a purge/recovery phase. The ratio between the maximum change of resistance (Δ*R*) and the baseline resistance (*R_0_*), after baseline correction with a Savitzky-Golay filter, was processed as the output. Figure 3 shows sample data of a DMAP-AuNP chemiresistor responding to three different tea aromas, as well as baseline resistance variation among the four investigated monolayer protected nanoparticles.

As shown in Figure 3A for a selected sensor element assembled with DMAP-AuNP, the sensor responses are strongest toward Earl Grey (2.57 ± 0.07%), followed by Bengal Spice^®^ (1.75 ± 0.08%) and Matcha (0.83 ± 0.05%). Fast and reversible detection was demonstrated with a sampling rate of 20 ms between data points. A feature of nanoparticle-based chemiresistor arrays is the capability of the system to respond to a variety of analytes with different signal strengths. This is achieved by variations in the chemistry of the monolayer protected nanoparticles as well as nanoparticle assembly morphology [20,30,42]. From a microscopic view, tea aromas full of many chemical ingredients interact at the molecular scale with the monolayer protected nanoparticle organic shells, which induces a change in interparticle separation that affects electron transport through the nanoparticle films [18,30,31]. Figure 3B illustrates baseline resistances and sensor responses to Black Cherry Berry for four different monolayer protected nanoparticles on a single chip. After eliminating devices with out-of-criterion baseline resistance, 38 of 48 devices remained valid for analysis. For each monolayer protected nanoparticle, sensor response measurements were carried out with at least eight individual sensing elements. Error bars reflect the spread of *R_0_* and Δ*R*/*R_0_* for each grouping of monolayer protected nanoparticles. Sensor elements assembled with aqueous-phase DMAP-AuNPs possess the lowest baseline resistance (6.8 ± 4.8 × 10^5^ Ω), followed by ODA-AuNPs (4.1 ± 2.4 × 10^6^ Ω), MPA-AuNPs (4.1 ± 2.2 ×10^7^ Ω), and ATP-AuNPs (4.9 ± 2.2 × 10^7^ Ω). Sensor responses follow the same trend as baseline resistance up to 10^6^ Ω range, after which responses stabilize around Δ*R*/*R_0_* = 5%.

The variations of baseline resistance and sensor response levels are essential for pattern recognition using machine learning algorithms. Depending on the effective chemical interactions between analytes and monolayer protected core-shell nanostructures, the AuNP layers swell at different magnitudes [43], which results in variations of sensor responses. The sorption of vapors into the sensing materials can be qualitatively understood in terms of non-covalent van der Waals interactions that vary with each analyte and sensor material [18,44,45].

### 3.2. Sensor Performance and Classification Accuracy

**Figure 4:**
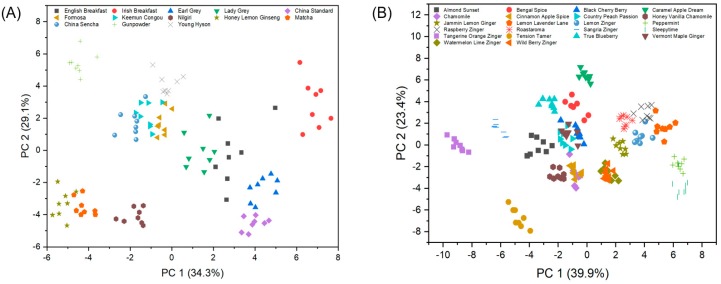
PCA plots of (**A**) eight black teas and five green teas, and (**B**) 22 herbal teas analyzed with the same sensor chip using four types of monolayer protected nanoparticles.

Sensor element responses (Δ*R*/*R_0_*) to the 35 different tea aromas were collected and processed as inputs for statistical analysis. Principal component analysis (PCA) was performed as an unsupervised method to examine the natural grouping and separations between the different teas. The analytes were analyzed in two separate groups. The first group consisted of the eight black teas and five green teas, and the second group comprised all 22 herbal teas. Figure 4 shows PCA plots of both groups to reveal successful separation and clustering of the data. In Figure 4A it is apparent that the green tea analytes (Honey Lemon Ginseng, Matcha, China Sencha, Young Hyson, and Gunpowder) all have PC1 scores <0, while most of the black teas have PC1 scores >0, with the exception of Formosa, Keemun congou, and Nilgiri. The latter, in particular, is located closer to some of the green teas. This can be partly explained by the texture of tea leaves, as the Nilgiri tea sample consists of fine powders, which was similar to both Honey Lemon Ginseng and Matcha. Overall, the PC1 separation suggests intrinsic differences between most of the green and black teas. Figure 4B shows more overlap of the herbal teas, which may be due to the similarity of ingredients. For instance, both Watermelon Lime Zinger^®^ and Wild Berry Zinger^®^ have hibiscus and rosehips listed as the top two ingredients, which can explain the overlap in PCA scores. The separation of clusters is limited by the 2D plot that only captures 63% of the variance in PCs 1 & 2.

**Figure 5:**
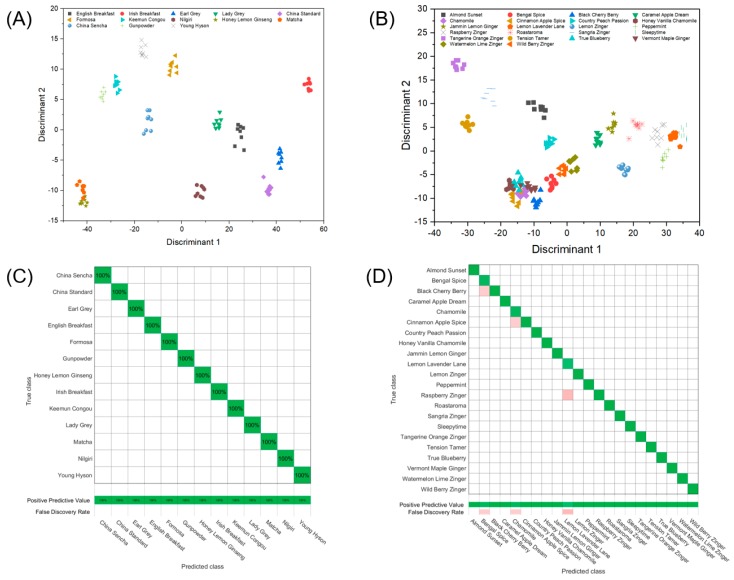
LDA plots of (**A**) eight black tea analytes and five green tea analytes and (**B**) 22 herbal tea analytes on the same chip assembled with four types of functionalized gold nanoparticles (38 devices). Confusion matrix shows (**C**) 100% classification accuracy for black teas and green teas, and 97.7% for (**D**) herbal teas.

As PCA only captures a qualitative account of aroma classification, supervised multivariate and machine learning models were used to provide a quantitative analysis [46,47]. One of the most robust classification techniques is linear discriminant analysis (LDA), which characterizes the input data based on linear combinations of sensor responses, and maximizes the distance between different classes [48]. Five-fold cross-validation was applied to all classification analyses. Specifically, the data entries were randomly divided into five groups. Among the five groups, four were used in the training set, and the remaining group was tested. The process rotated five times so that every data entry had been used in the testing set. For the first group of analytes (black teas and green teas), out of 104 measurements, the training size was 83 and the testing size was 21 for each rotation. For the second group (herbal teas), out of 176 measurements, the training size was 141 and the testing size was 35 for each rotation. Figure 5 shows an LDA plot and confusion matrix for both groups of analytes.

As shown in Figure 5, 100% classification accuracy was achieved for green and black teas, and 97.7% accuracy was achieved for herbal teas, where confusion occurs three times. For example, one of the misclassifications is treating Raspberry Zinger^®^ as Lemon Levander Lane^TM^. This can be rationalized from the PCA plot where close clustering is observed for these two classes. If the teas are analyzed as a single group of 35, the LDA classification accuracy is 98.6%.

Table 2 summarizes the LDA classification accuracy for a full sensor chip, as well as a comparison of classification accuracy of the four separate monolayer protected nanoparticles used in this study. The full sensor chip with all 38 sensing elements is obviously better than any of the separate chemistries alone, although the DMAP chemistry does very well with the green and black teas (97.1%). For a more even comparison, two devices from each sensing material were randomly selected to make a group of eight sensing elements for comparison with single particle data on an equal number basis (eight multi-particle vs. eight single particle). The result showed a 91.4 ± 3.3% accuracy for black and green teas, and 88.6 ± 3.7% for herbal teas. The combination of the four monolayer protected nanoparticles clearly performs better overall than any single type of nanoparticle, which supports a broader hypothesis that more chemical diversity can enhance sensor performance. We speculate that adding more of each type of monolayer protected nanoparticle will also improve overall sensor performance.

### 3.3. Sensor Stability

**Figure 6:**
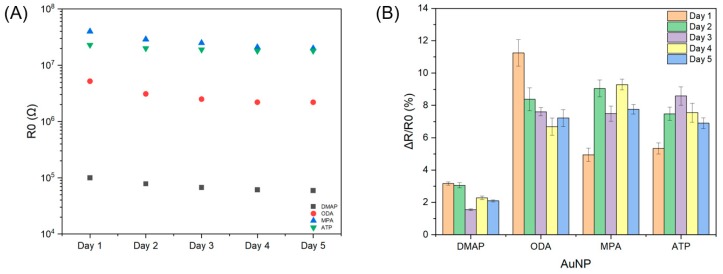
(**A**) Baseline resistance of four sensor elements with different monolayer protected nanoparticles on the same chip during a five-day experiment. (**B**) Sensing responses toward Irish Breakfast Tea.

To evaluate sensor stability, a five-day experiment was performed for the classification of black and green tea aromas. Firstly, the baseline resistance of each working device was monitored over time. Figure 6A shows a gradual decrease of baseline resistance, which was larger during Days 1–3 and flattened after Day 4. All sensing materials underwent a decrease in baseline resistance. Sensing profiles on Figure 6B show that for DMAP-AuNP and ODA-AuNP, there was a trend of decreasing sensitivity, but not for MPA-AuNP or ATP-AuNP. This is likely due to the difference in particle morphology changes during the reversible sensing process.

Besides linear discriminant analysis (LDA), two other types of classification algorithms were examined using MATLAB Classification Learner Toolbox, including support vector machine (SVM) and k-nearest neighbors (KNN). Again, five-fold cross-validation was applied for all methods. Table 3 shows the classification accuracy of black and green teas over five days for 38 sensing elements on a sensor chip. For all four methods, the classification accuracy declined at Day 3, however, it mostly recovered by Day 5. This may be related to changes of baseline resistance for Days 1–3. The baseline resistance stabilized at Day 5, which resulted in an improved classification accuracy. The Random Forest (RF) method is a relatively newer machine learning approach that has been successful in many other studies, therefore, we also tested it on our data set. The RF method performed very well for all days with >98% accuracy, but it also shows a small dip at Days 3 & 4.

## 4. Conclusions

In this project, we demonstrated gold nanoparticle based chemiresistor arrays in order to classify 35 types of tea beverages, including eight black teas, five green teas, and 22 herbal teas. Sensor performance was evaluated based on classification accuracy using linear discriminant analysis (LDA) and several machine learning algorithms. A classification accuracy higher than 97% was achieved for both green and black teas (group 1), and herbal teas (group 2). The results demonstrate more robust performance for sensor arrays assembled with multiple types of monolayer protected nanoparticles compared to arrays of a single type, which supports a broader hypothesis that more diverse chemistry can enhance sensor performance for resolving complex odors, including teas. Short term sensor stability was assessed by performing a five-day experiment using teas from group 1. During the five-day experiment, a classification accuracy higher than 90% was obtained and sensor performance recovered to initial performance after the fifth day. The findings suggest that nanoparticle sensor arrays can be useful for detection and discrimination of complex odors, including tea aromas.

## Figures and Tables

**Table 1:** Detailed descriptions of the investigated teas in this study.

Category	Flavor	Main Ingredient
Black tea	English Breakfast ^a^	Black tea
	Irish Breakfast ^a^	Black tea
	Earl Grey ^a^	Black tea, bergamot flavor
	Lady Grey ^a^	Black tea, orange peel, lemon peel
	China Standard ^b^	Black tea
	Formosa ^b^	Black tea
	Keemun Congou ^b^	Black tea
	Nilgiri ^b^	Black tea
	Honey Lemon Ginseng ^c^	Green tea, white tea, eleuthero
	Matcha ^c^	Green tea, organic matcha
Green tea	China Sencha ^b^	Green tea
	Gunpowder ^b^	Green tea
	Young Hyson ^b^	Green tea
Herbal tea	Almond Sunset^TM c^	Roasted carob, roasted barley, roasted chicory
	Bengal Spice^® c^	Cinnamon, roasted chicory, roasted carob
	Black Cherry Berry ^c^	Hibiscus, rosehips, roasted chicory
	Caramel Apple Dream^® c^	Cinnamon, hibiscus, natural caramel and apple
	Chamomile ^c^	Chamomile
	Cinnamon Apple Spice ^c^	Cinnamon, hibiscus, chamomile
	Country Peach Passion^® c^	Orange peel, rosehips, hawthorn
	Honey Vanilla Chamomile ^c^	Chamomile, orange peel, natural honey flavor
	Jammin’ Lemon Ginger ^c^	Ginger, lemon verbena, lemongrass
	Lemon Lavender Lane^TM c^	Lemongrass, lemon verbena, lavender
	Lemon Zinger^® c^	Hibiscus, rosehips, roasted chicory
	Peppermint ^c^	Peppermint
	Raspberry Zinger^® c^	Hibiscus, rosehips, roasted chicory
	Roastaroma^® c^	Roasted barley, roasted chicory, roasted carob
	Sangria Zinger^® c^	Hibiscus, rosehips, orange peel
	Sleepytime^® c^	Chamomile, spearmint, lemongrass
	Tangerine Orange Zinger^® c^	Hibiscus, rosehips, blackberry leaves
	Tension Tamer^® c^	Eleuthero, peppermint, cinnamon
	True Blueberry^® c^	Hibiscus, rosehips, orange peel
	Vermont Maple Ginger^TM c^	Ginger, cinnamon, natural maple flavor
	Watermelon Lime Zinger^® c^	Hibiscus, rosehips, orange peel
	Wild Berry Zinger^® c^	Hibiscus, rosehips, roasted chicory

^a^ Purchased from Twinnings; ^b^ purchased from Solstice Tea Traders; ^c^ purchased from Celestial Seasonings.

**Table 2:** Classification accuracy (LDA) comparison among different AuNP sensing elements.

AuNP	Number of Working Devices	Accuracy (%)	
		Black and Green Tea	Herbal Tea
DMAP	10	97.1	75.0
ODA	11	77.9	77.8
MPA	8	52.9	52.8
ATP	9	53.8	43.8
Overall	38	100	97.7

**Table 3:** Sensor stability analysis for eight black teas and five green teas.

Day	LDA (%)	SVM (%)	KNN (%)	RF (%)
1	100	97.1	93.3	99.7
2	96.2	97.1	98.1	99.7
3	90.4	88.5	87.5	98.6
4	86.5	91.3	88.5	98.9
5	97.1	98.1	97.1	99.8

## References

[1] Weisburger J.H. (1997). Tea and health: A historical perspective. Cancer Lett..

[2] Ravikumar C. (2014). Review on herbal teas. J. Pharm. Sci. Res..

[3] Aoshima H., Hirata S., Ayabe S. (2007). Antioxidative and anti-hydrogen peroxide activities of various herbal teas. Food Chem..

[4] Kumar A., Nair A.G.C., Reddy A.V.R., Garg A.N. (2005). Analysis of essential elements in Pragya-peya—A herbal drink and its constituents by neutron activation. J. Pharm. Biomed. Anal..

[5] Nash L.A., Ward W.E. (2017). Tea and bone health: Findings from human studies, potential mechanisms, and identification of knowledge gaps. Crit. Rev. Food Sci. Nutr..

[6] Sanlier N., Gokcen B.B., Altuğ M. (2018). Tea consumption and disease correlations. Trends Food Sci. Technol..

[7] Xing L., Zhang H., Qi R., Tsao R., Mine Y. (2019). Recent advances in the understanding of the health benefits and molecular mechanisms associated with green tea polyphenols. J. Agric. Food Chem..

[8] McKay D.L., Blumberg J.B. (2006). A review of the bioactivity and potential health benefits of chamomile tea (*Matricaria recutita* L.). Phyther. Res..

[9] McKay D.L., Blumberg J.B. (2006). A review of the bioactivity and potential health benefits of peppermint tea (*Mentha piperita* L.). Phyther. Res..

[10] Graboski A.M., Galvagni E., Manzoli A., Shimizu F.M., Zakrzevski C.A., Weschenfelder T.A., Steffens J., Steffens C. (2018). Lab-made electronic-nose with polyaniline sensor array used in classification of different aromas in gummy candies. Food Res. Int..

[11] Wang B., Han J., Bender M., Hahn S., Seehafer K., Bunz U.H.F. (2018). Poly(para-phenyleneethynylene)-sensor arrays discriminate 22 different teas. ACS Sens..

[12] Dasary S.S.R., Singh A.K., Senapati D., Yu H., Ray P.C. (2009). Gold nanoparticle based label-free SERS probe for ultrasensitive and selective detection of trinitrotoluene. J. Am. Chem. Soc..

[13] Banerjee M.B., Roy R.B., Tudu B., Bandyopadhyay R., Bhattacharyya N. (2019). Black tea classification employing feature fusion of E-Nose and E-Tongue responses. J. Food Eng..

[14] Fu K., Willis B.G. (2015). Characterization of DNA as a solid-state sorptive vapor sensing material. Sens. Actuators B Chem..

[15] Saha K., Agasti S.S., Kim C., Li X., Rotello V.M. (2012). Gold nanoparticles in chemical and biological sensing. Chem. Rev..

[16] Dutta D., Ghosh S., Narjinary M., Bhattacharyya N., Bandyopadhyay R. (2016). Tin oxide based gas sensor array in electronic nose to monitor aroma of black tea. Sens. Lett..

[17] Chen Q., Zhao J., Chen Z., Lin H., Zhao D.A. (2011). Discrimination of green tea quality using the electronic nose technique and the human panel test, comparison of linear and nonlinear classification tools. Sens. Actuators B Chem..

[18] Potyrailo R.A. (2017). Toward high value sensing: Monolayer-protected metal nanoparticles in multivariable gas and vapor sensors. Chem. Soc. Rev..

[19] Peveler W.J., Yazdani M., Rotello V.M. (2016). Selectivity and specificity: Pros and cons in sensing. ACS Sens..

[20] Hubble L.J., Cooper J.S., Sosa-Pintos A., Kiiveri H., Chow E., Webster M.S., Wieczorek L., Raguse B. (2015). High-throughput fabrication and screening improves gold nanoparticle chemiresistor sensor performance. ACS Comb. Sci..

[21] Kahn N., Lavie O., Paz M., Segev Y., Haick H. (2015). Dynamic nanoparticle-based flexible sensors: Diagnosis of ovarian carcinoma from exhaled breath. Nano Lett..

[22] Wohltjen H., Snow A.W. (1998). Colloidal metal-insulator-metal ensemble chemiresistor sensor. Anal. Chem..

[23] Dovgolevsky E., Konvalina G., Tisch U., Haick H. (2010). Monolayer-capped cubic platinum nanoparticles for sensing nonpolar analytes in highly humid atmospheres. J. Phys. Chem. C.

[24] Moreno M., Ibañez F.J., Jasinski J.B., Zamborini F.P. (2011). Hydrogen reactivity of palladium nanoparticles coated with mixed monolayers of alkyl thiols and alkyl amines for sensing and catalysis applications. J. Am. Chem. Soc..

[25] Gao T., Wang Y., Luo Y., Zhang C., Pittman Z., Oliveira A., Craig H., Zhao J., Willis B.G. (2018). Fast and reversible chemiresistive sensors for robust detection of organic vapors using oleylamine-functionalized palladium nanoparticles. Int. J. High Speed Electron. Syst..

[26] Fu K., Li S., Jiang X., Wang Y., Willis B.G. (2013). DNA gold nanoparticle nanocomposite films for chemiresistive vapor sensing. Langmuir.

[27] Fu K., Pedrick W., Wang H., Lamarche A., Jiang X., Willis B.G., Li S., Wang Y. Polynucleotide-functionalized gold nanoparticles as chemiresistive vapor sensing elements. Proceedings of the IEEE Sensors.

[28] Krasteva N., Guse B., Besnard I., Yasuda A., Vossmeyer T. (2003). Gold nanoparticle/PPI-dendrimer based chemiresistors—Vapor-sensing properties as a function of the dendrimer size. Sens. Actuators B Chem..

[29] Choi J.P., Coble M.M., Branham M.R., DeSimone J.M., Murray R.W. (2007). Dynamics of CO_2_-plasticized electron transport in au nanoparticle films: Opposing effects of tunneling distance and local site mobility. J. Phys. Chem. C.

[30] Fu K., Chen S., Zhao J., Willis B.G. (2016). Dielectrophoretic assembly of gold nanoparticles in nanoscale junctions for rapid, miniature chemiresistor vapor sensors. ACS Sens..

[31] Ibañez F.J., Zamborini F.P. (2012). Chemiresistive sensing with chemically modified metal and alloy nanoparticles. Small.

[32] Brust M., Walker M., Bethell D., Schiffrin D.J., Whyman R. (1994). Synthesis of thiol-derivatised gold nanoparticles in a two-phase liquid-liquid system. J. Chem. Soc. Chem. Commun..

[33] Gittins D.I., Caruso F. (2001). Spontaneous phase transfer of nanoparticulate metals from organic to aqueous media. Angew. Chem. Int. Ed..

[34] Chen S., Jenkins S.V., Tao J., Zhu Y., Chen J. (2013). Anisotropic seeded growth of Cu-M (M = Au, Pt, or Pd) bimetallic nanorods with tunable optical and catalytic properties. J. Phys. Chem. C.

[35] Garg N., Mohanty A., Lazarus N., Schultz L., Rozzi T.R., Santhanam S., Weiss L., Snyder J.L., Fedder G.K., Jin R. (2010). Robust gold nanoparticles stabilized by trithiol for application in chemiresistive sensors. Nanotechnology.

[36] Cooper J.S., Raguse B., Chow E., Hubble L., Müller K.H., Wieczorek L. (2010). Gold nanoparticle chemiresistor sensor array that differentiates between hydrocarbon fuels dissolved in artificial seawater. Anal. Chem..

[37] Mohamed A.A., Salmi Z., Dahoumane S.A., Mekki A., Carbonnier B., Chehimi M.M. (2015). Functionalization of nanomaterials with aryldiazonium salts. Adv. Colloid Interface Sci..

[38] Orefuwa S.A., Ravanbakhsh M., Neal S.N., King J.B., Mohamed A.A. (2014). Robust organometallic gold nanoparticles. Organometallics.

[39] Overton A.T., Mohamed A.A. (2012). Gold(III) diazonium complexes for electrochemical reductive grafting. Inorg. Chem..

[40] Goss C.A., Charych D.H., Majda M. (1991). Application of (3-mercaptopropyl)trimethoxysilane as a molecular adhesive in the fabrication of vapor-deposited gold electrodes on glass substrates. Anal. Chem..

[41] Barsotti R.J., Vahey M.D., Wartena R., Chiang Y.M., Voldman J., Stellacci F. (2007). Assembly of metal nanoparticles into nanogaps. Small.

[42] Albert K.J., Lewis N.S., Schauer C.L., Sotzing G.A., Stitzel S.E., Vaid T.P., Walt D.R. (2000). Cross-reactive chemical sensor arrays. Chem. Rev..

[43] Kummer A.M., Hierlemann A., Baltes H. (2004). Tuning sensitivity and selectivity of complementary metal oxide semiconductor-based capacitive chemical microsensors. Anal. Chem..

[44] Grate J.W., Nelson D.A., Skaggs R. (2003). Sorptive behavior of monolayer-protected gold nanoparticle films: Implications for chemical vapor sensing. Anal. Chem..

[45] Grate J.W., Patrash S.J., Abraham M.H. (1995). Method for estimating polymer-coated acoustic wave vapor sensor responses. Anal. Chem..

[46] Bro R., Smilde A.K. (2014). Principal component analysis. Anal. Methods.

[47] Nallon E.C., Schnee V.P., Bright C., Polcha M.P., Li Q. (2016). Chemical discrimination with an unmodified graphene chemical sensor. ACS Sens..

[48] Jurs P.C., Bakken G.A., McClelland H.E. (2000). Computational methods for the analysis of chemical sensor array data from volatile analytes. Chem. Rev..

